# Integration of network pharmacology and molecular docking to explore the molecular mechanism of Cordycepin in the treatment of Alzheimer’s disease

**DOI:** 10.3389/fnagi.2022.1058780

**Published:** 2022-12-23

**Authors:** Xiaoying Ma, Ying Zhao, Tao Yang, Na Gong, Xun Chen, Guoli Liu, Jun Xiao

**Affiliations:** The Institute of Edible Fungi, Liaoning Academy of Agricultural Sciences, Shenyang, China

**Keywords:** network pharmacology, molecular docking, Cordycepin, Alzheimer’s disease, target

## Abstract

**Background:**

Cordycepin is a nucleoside adenosine analog and an active ingredient isolated from the liquid fermentation of *Cordyceps*. This study sought to explore the mechanism underlying the therapeutic effect of Cordycepin against Alzheimer’s disease using network pharmacology and molecular docking technology.

**Methods:**

TCMSP, SYMMAP, CTD, Super-pred, SEA, GeneCards, DisGeNET database, and STRING platform were used to screen and construct the target and protein interaction network of Cordycepin for Alzheimer’s disease. The results of Gene Ontology annotation and KEGG pathway enrichment analysis were obtained based on the DAVID database. The Omicshare database was also applied in GO and KEGG pathway enrichment analysis of the key targets. The protein–protein interaction network was constructed using the STRING database, and the potential effective targets for AD were screened based on the degree values. The correlation between the potential targets of Cordycepin in the treatment of AD and APP, MAPT, and PSEN2 was analyzed using (GEPIA) databases. We obtained potential targets related to aging using the Aging Altas database. Molecular docking analysis was performed by AutoDock Vina and Pymol software. Finally, we validated the significant therapeutic targets in the Gene Expression Omnibus (GEO) database.

**Results:**

A total of 74 potential targets of Cordycepin for treating Alzheimer’s disease were identified. The potential targets of Cordycepin for the treatment of AD mainly focused on Lipid and atherosclerosis (hsa05417), Platinum drug resistance (hsa01524), Apoptosis (hsa04210), and Pathways in cancer (hsa05200). Our findings suggest that the therapeutic effect of Cordycepin on AD is primarily associated with these biological processes. We obtained 12 potential therapeutic targets for AD using the degree value in Cytoscape. Interestingly, AKT1, MAPK8, BCL2L1, FOXO3, and CTNNB1 were not only significantly associated with pathogenic genes (APP, MAPT, and PSEN2) but also with longevity in Alzheimer’s Disease. Thus we speculated that the five target genes were potential core targets mediating the therapeutic effect of Cordycepin against AD. Moreover, molecular docking results analysis showed good binding affinity between Cordycepin and the five core targets. Overall, MAPK8, FOXO3 and CTNNB1 may have significant clinical and treatment implications.

**Conclusion:**

Network pharmacology demonstrated that Cordycepin exerts a therapeutic effect against Alzheimer’s disease *via* multiple targets and signaling pathways and has huge prospects for application in treating neurodegenerative diseases.

## Introduction

In recent years, the prevalence of geriatic diseases has significantly increased in China due to population aging. According to the latest Alzheimer’s disease (AD) International (ADI) data documented in the World Alzheimer’s Report 2021, there are about 55 million dementia patients in the world, and it is expected that by 2030, this number will reach 78 million (McGill University Alzheimer’s disease international, World Alzheimer Report 2021, Journey through the diagnosis of dementia, 2021).

Alzheimer’s disease is a progressive neurodegenerative disease related to aging (Holtzman and Ulrich, 2019). It is widely acknowledged that clinical characteristics of Alzheimer’s disease, including cognitive decline, memory disorders, aphasia, apraxia, and agnosia, severely affect patient lives (Zhang and Zheng, 2019). AD is a complex neurodegenerative disorder with various pathological factors. Although the past decade has witnessed unprecedented progress in understanding AD, more basic research is warranted. So far, there are still no related effective drugs to cure AD patients. Notwithstanding that many drugs have been developed for treating central nervous system diseases, they have significant limitations, including severe side effects and complications (Govindula et al., 2021). Currently available treatments, based on early findings of cholinergic deficit, have only provided limited improvement in cognitive functions in AD. It is widely thought that previous studies have been largely ineffective given that they only targeted a single aspect or mechanism of the disease, which is often caused by the interplay of different factors (Ju and Tam, 2020, 2021). Therefore, considering the complexity of AD pathogenesis, designing multifunctional drugs exhibiting multitarget potential may lead to better results in developing AD treatments (Ju and Tam, 2020).

**Figure 1:**
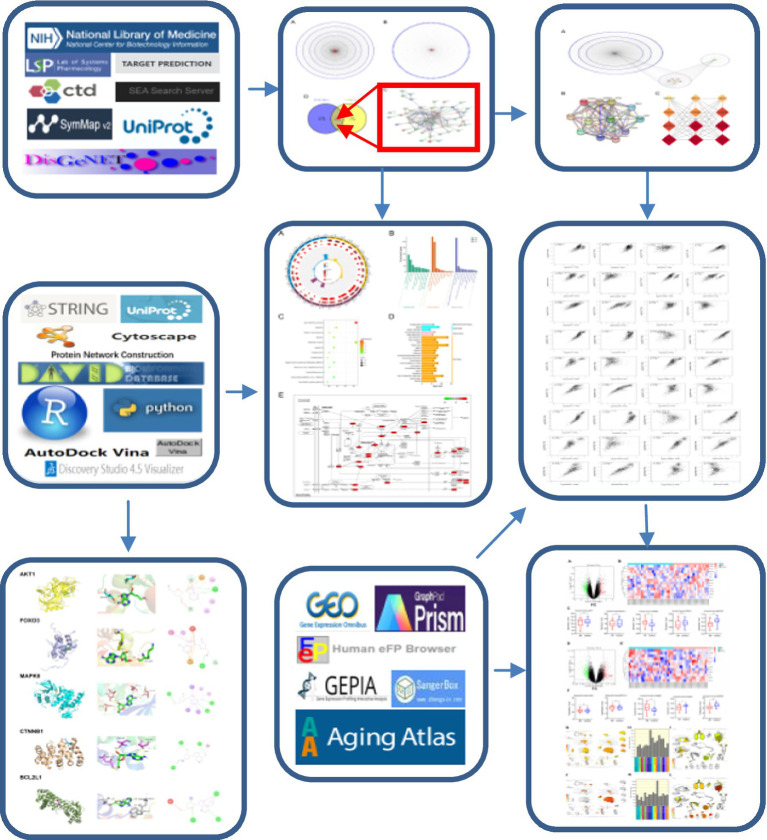
Network Pharmacology Workflow of Cordycepin in the treatment of Alzheimer’s Disease.

Cordycepin is a nucleoside adenosine analog and an active ingredient isolated from the liquid fermentation of *Cordyceps* (a precious medicinal mushroom). Cordycepin exhibits a wide range of biological activities and has an enormous impact in many therapeutic research areas (Yang et al., 2019). *Cordyceps Sinensis* is one of the most widely used medicinal fungi and has various positive pharmacological effects (Elkhateeb et al., 2019). It exerts a protective effect against senescence, inhibits fatigue, and exerts antioxidant, anti-inflammatory, anticancer, antihepatotoxic, anti-fibrotic and neuroprotective effects (Qin et al., 2019). It has been reported that Cordycepin can treat neurodegenerative diseases by inhibiting the expression of iNOS, Akt, MAPKs, NF-κB, and COX-2 in microglial cells (Jeong et al., 2010). Besides, it has been found that Cordycepin can restore Lipopolysaccharide (LPS)-induced decrease of primary hippocampal neurons and enhance cell viability and neuronal differentiation (Peng et al., 2015). Indeed, it is challenging to study the anti-Alzheimer’s effect of Cordycepin based on traditional Chinese medicine pharmacology; however, studying pharmacological mechanisms using network pharmacology makes it feasible (Zhai et al., 2018). Importantly, network pharmacology analysis can elucidate the mechanism of drugs and provides an effective method for developing traditional Chinese medicine through the nodes and edges in the biological network. In this regard, network pharmacology can screen active ingredients and potential therapy targets, elucidate the complex mechanisms by which drugs and prescriptions treat diseases, and reveal medicinal properties. The combination of TCM database and computer software analysis not only expands TCM knowledge, but also greatly promotes the internationalization of TCM. In this study, the anti-Alzheimer disease targets of Cordycepin were retrieved by network pharmacology (Figure 1). PPI, gene ontology, and KEGG pathway analyses were carried out, the correlation between the potential target and disease-related genes was analyzed, and screening of potential targets related to aging was conducted using Aging Altas. Molecular docking verification of the core target was carried out, and the clinical significance of the core target was evaluated in the GEO database to elucidate the mechanisms underlying the therapeutic effect of Cordycepin against Alzheimer’s disease.

## Materials and methods

### Screening of Cordycepin targets

The biological targets of cordycepin were obtained from TCMSP,^1^ Pubmed,^2^ SYMMAP,^3^ CTD,^4^ Super-pred (prediction. Charite.de) (probability >80%), and SEA (https://sea.bkslab.org/; z score > 30). Cordycepin target information was generated by entering “Cordycepin” in these databases, searching for cordycepin targets, and selecting its default option in parameter settings. We merged the results of the six databases and removed duplicate targets to obtain targets of Cordycepin.

### Determination of the targets of Cordycepin in Alzheimer’s disease

The targets of Alzheimer’s disease were obtained from Genecards,^5^ DisGeNT,^6^ and CTD^7^ databases. Similarly, we translated the AD target proteins ID into the corresponding gene symbol by UniProt ID mapping tools. Then, we obtained the intersection of gene targets of Cordycepin and Alzheimer’s disease using Venny^8^ online tools. The intersected targets were further analyzed to obtain candidate targets for AD treatment. The study was conducted in accordance with the tenets of the Declaration of Helsinki (as revised in 2013).

### Enrichment analysis of the intersected genes

To better understand the functions of the intersected targets, gene ontology (GO) and KEGG enrichment analysis were carried out using the DAVID.^9^ For GO enrichment analysis and KEGG analysis, GO terms and KEGG pathways with *p*-values <0.05 were considered significantly enriched. The results of pathway enrichment analysis were plotted using the bioinformatics^10^ online platform.

### Construction of the drug-ingredient-target network (including protein–protein interactions) and pathways

STRING^11^ is an online biological database that can predict protein–protein interaction (PPI) networks. We used common targets Cordycepin against Alzheimer’s disease as inputs to a STRING database, obtaining complete PPI networks and related data. To describe the mechanisms of Cordycepin against Alzheimer’s disease, a network of components-disease-targets-pathways was constructed using Cytoscape software (3.9.1) based on the active constituents, corresponding targets, and pathway information. Components, targets, pathways, and diseases are represented as nodes, and connections between proteins are represented as edges.

### Correlation analysis between key targets and key genes of AD

To confirm the relationship between key genes and major causative gene amyloidprecursor protein (APP) or microtubule-associated protein tau (MAPT) and presenilin2 (PSEN2), gene correlation analysis was conducted using GEPIA. Key genes and APP, MAPT and PSEN2 were input into the search words in turn, and all brain tissues were selected from GTEx tissues for correlation analysis.AgingAtlas is a bioinformatics tool used for assessing the genetic correlation between Ageing and Longevity (Liu et al., 2020). It is well-established that AD is an aging-related disease. The aging-associated genes in this study were obtained from the Aging Altas. We selected genes that were not only aging-related but also significantly associated with AD.

### Molecular docking

The screened key genes served as the key targets for receptors, and the information on receptor structures was obtained from PDB^12^ and the Uniprot database. The 2D structures of Cordycepin were obtained from TSCMP databases, and optimized ligands were used as starting points for docking. The protein-ligand molecular docking and docking calculations study was performed with AutoDock Vina. We obtained the conformation and the lowest binding energy of ligand-receptor interactions after the docking search was completed. Finally, the receptor-ligand binding image was visualized using Pymol software.

### Clinical characteristics and tissue enrichment of key targets

To obtain clinical implications of potential key targets, we searched transcriptome data for normal control and AD patient brain tissues in the GEO database (http://www.ncbi.nlm.nih.gov/geo/). Differentially expression analysis of AD-related genes was conducted between control and AD patients using the GEO2R tool. Graphs were generated using GraphPad Prism 8. Then, the expression distribution of key targets in neural typical was analyzed using the Human eFP Browser (http://bar.utoronto.ca/efp_human/; Patel et al., 2016), and the distribution of key genes in the whole body was obtained. The distribution of key gene expression was researched in the whole body of mice using the mouse EFP browser.^13^

## Result

### The intersected targets of Cordycepin and Alzheimer’s disease

**Figure 2:**
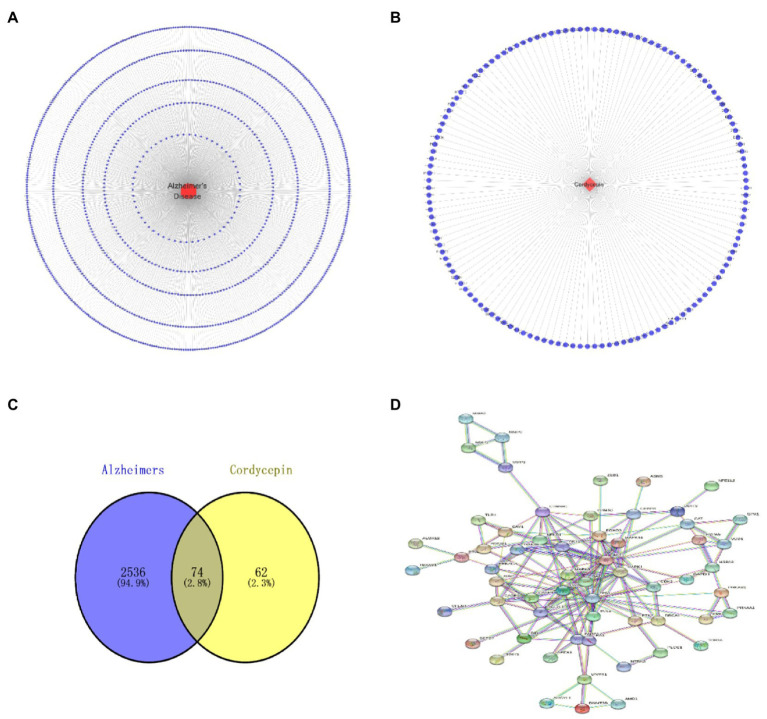
Alzheimer’s disease target network diagram **(A)**; Cordycepin related target network diagram **(B)**; Cordycepin-anti-Alzheimer disease target PPI network diagram **(C)**; Cordycepin-anti-Alzheimer disease Venn plot **(D)**.

Based on TCMSP, Pubmed, SYMMAP, CTD, Super-pred and SEA, we obtained 136 putative targets of Cordycepin. After retrieval from the DisGeNet, CTD, and GeneCards databases, 2,848 AD-related targets were obtained based on an Inference Score > 30. The intersection of Cordycepin targets and the Alzheimer’s disease targets was visualized in a Venn plot, yielding 74 putative targets for treating AD (Figure 2D).

### Go and KEGG pathway enrichment analysis

**Figure 3:**
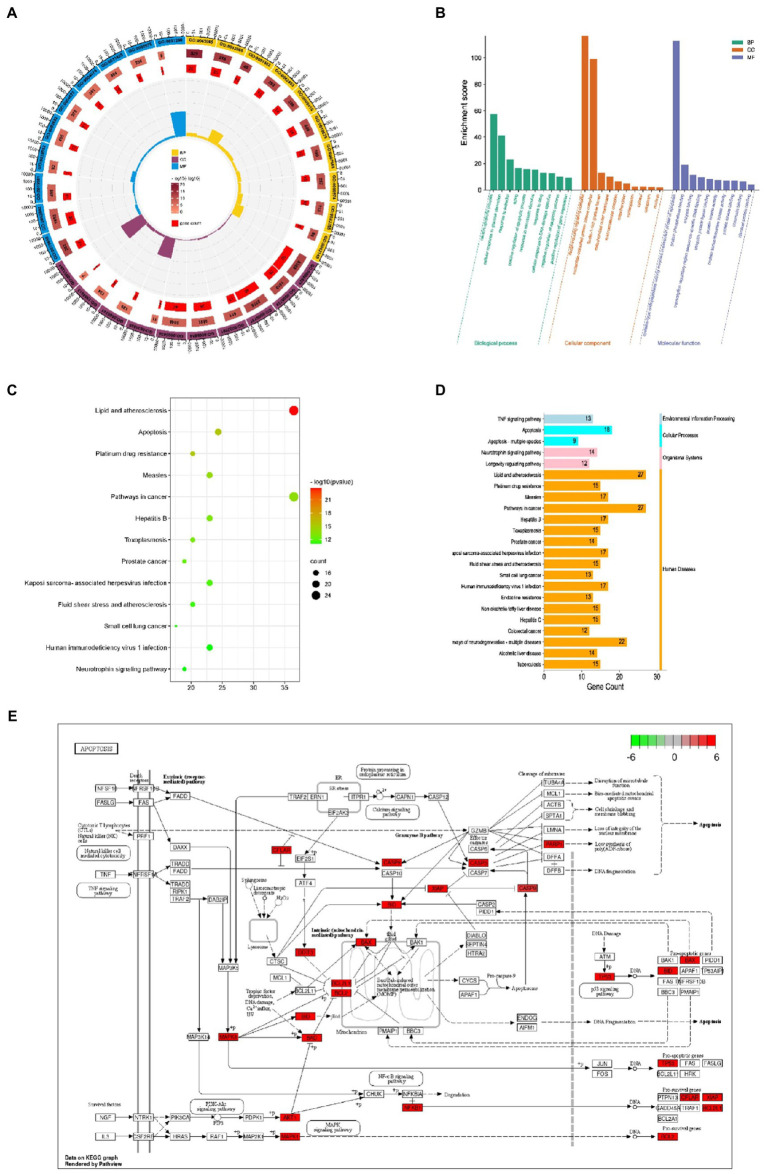
GO annotation of the Cordycepin-anti-Alzheimer disease targets **(A,B)**; KEGG pathway analysis of Cordycepin-anti-Alzheimer disease targets **(C,D)**; apoptosis pathway **(E)**. The red mark represents enrichment of the target of Cordycepin in the apoptosis pathway.

74 intersection genes were uploaded to DAVID for GO/KEGG analyses, and the significance level was set at *p* ≤ 0.001 (***). A total of 152 GO biological process (BP), 12 GO cellular component (CC), 30 GO molecular function (MF) and 96 KEGG pathways were significantly enriched. As shown in Figures 3A,B, significantly enriched biological processes mainly included positive and negative regulation of apoptosis, neuron apoptosis and drug response. Significantly enriched GO terms in the molecular function category included ‘enzyme activity’, ‘protease binding’ and ‘transcription factor binding’. Cellular components (CC) terms included nucleus, macromolecular complex and mitochondria. The enriched GO items were also associated with neuronal apoptosis, death-induced signal complex formation, and cysteine-type endopeptidase activity involved in the apoptotic process. Taken together, our results indicate that the effect of Cordycepin on AD is primarily associated with these biological processes.

### KEGG pathway analysis

KEGG analysis yielded a total of 96 pathways, and the top 13 enriched pathways are shown in Figures 3C,D. The potential targets in Cordycepin treatment of AD disease mainly focused on lipids arteriosclerosis (hsa05417), Platinum drug resistance (hsa01524), Apoptosis (hsa04210), Pathways in Cancer (hsa05200). Taken together, these results imply that Cordycepin may exert anti-AD effects by binding to cysteine-type endopeptidase in mitochondria from the caspase family that plays an essential role in apoptosis, and then inhibiting the formation of the death-inducing signaling complex of endopeptidases *via* the death domain protein recruitment, which in turn affects the occurrence of apoptosis, slowing neuronal cell death.

### Screening cordycepin-anti-Alzheimer disease key targets and target interaction network.

**Figure 4:**
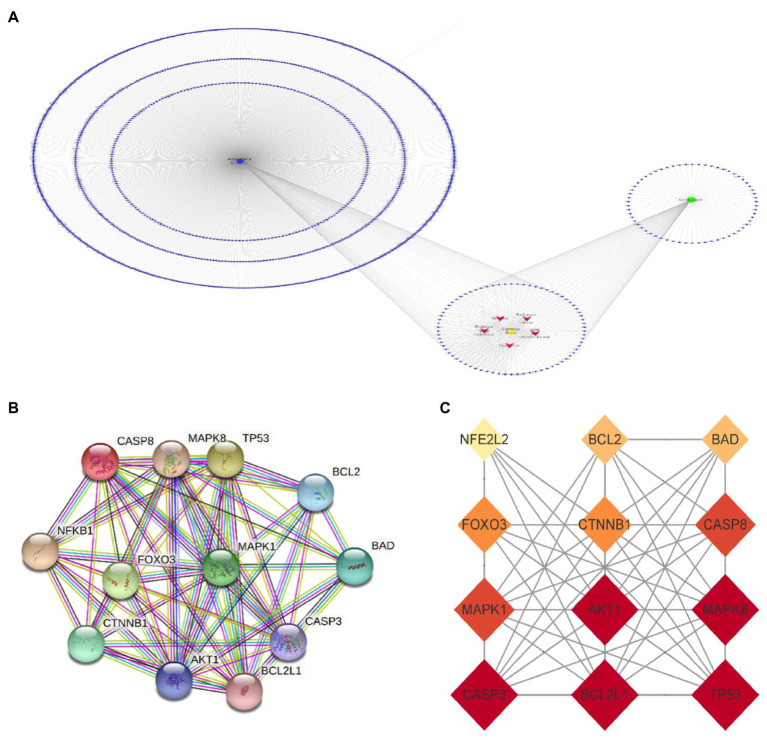
Cordycepin-Alzheimer disease-pathway target network **(A)**; 12 key targets of Cordycepin-anti-Alzheimer PPI network **(B,C)**.

The names of cordycepin-target and Alzheimer’s disease-target genes were converted into gene ID based on the UniProt database. Subsequently, we selected the intersected target genes between cordycepin-targets and Alzheimer’s disease-related targets using Venn plots. The intersected target genes were analyzed with the STRING database^14^ and imported interactions with a confidence score ≥ 0.9 for visualization. Next, a topological analysis of gene–gene network graphs was performed; 12 genes were selected as the Cordycepin-anti-Alzheimer key targets according to the rank node degree scores. Then, a protein–protein interaction (PPI) network of the 12 enrichment genes was constructed by the STRING database, then core targets including TP53, AKT1, CASP3, BCL2L1, MAPK8, MAPK1, CASP8, CTNNB1, FOXO3, BAD, BCL2, and NFE2L2 were obtained (Figure 4).

### Analysis of Core targets based on GEPIA

**Figure 5:**
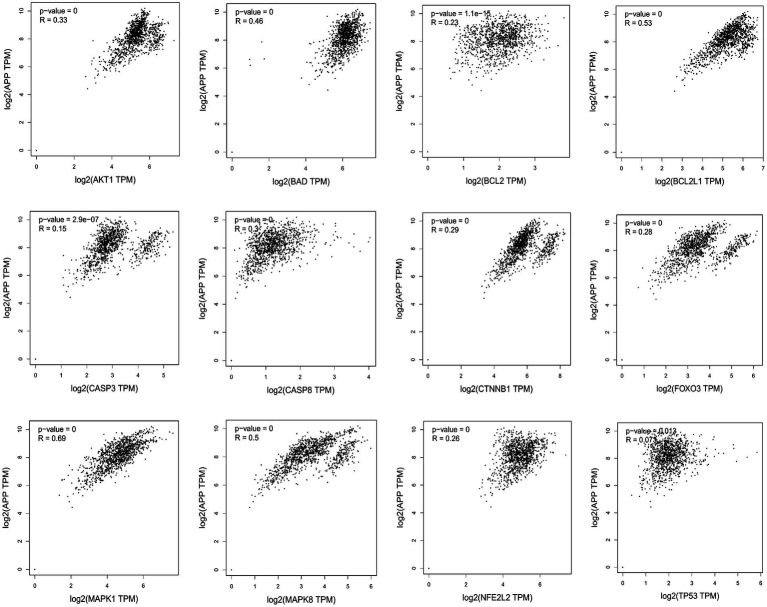
Analysis of the correlation between 12 key targets of Cordycepin and the pathogenic gene of Alzheimer’s disease APP.

**Figure 6:**
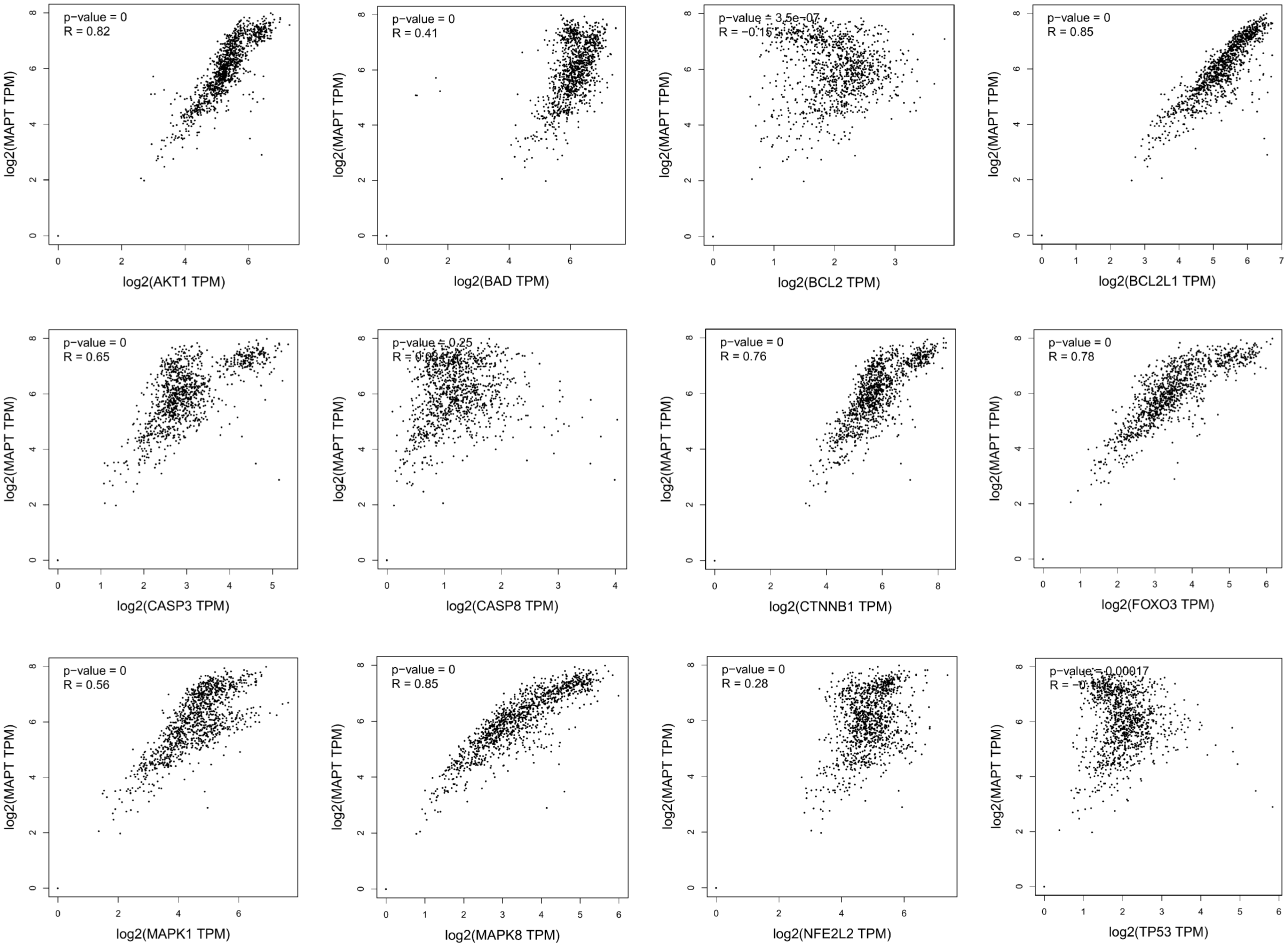
Analysis of the correlation between 12 key targets of Cordycepin and the pathogenic gene of Alzheimer’s disease MAPT.

**Figure 7:**
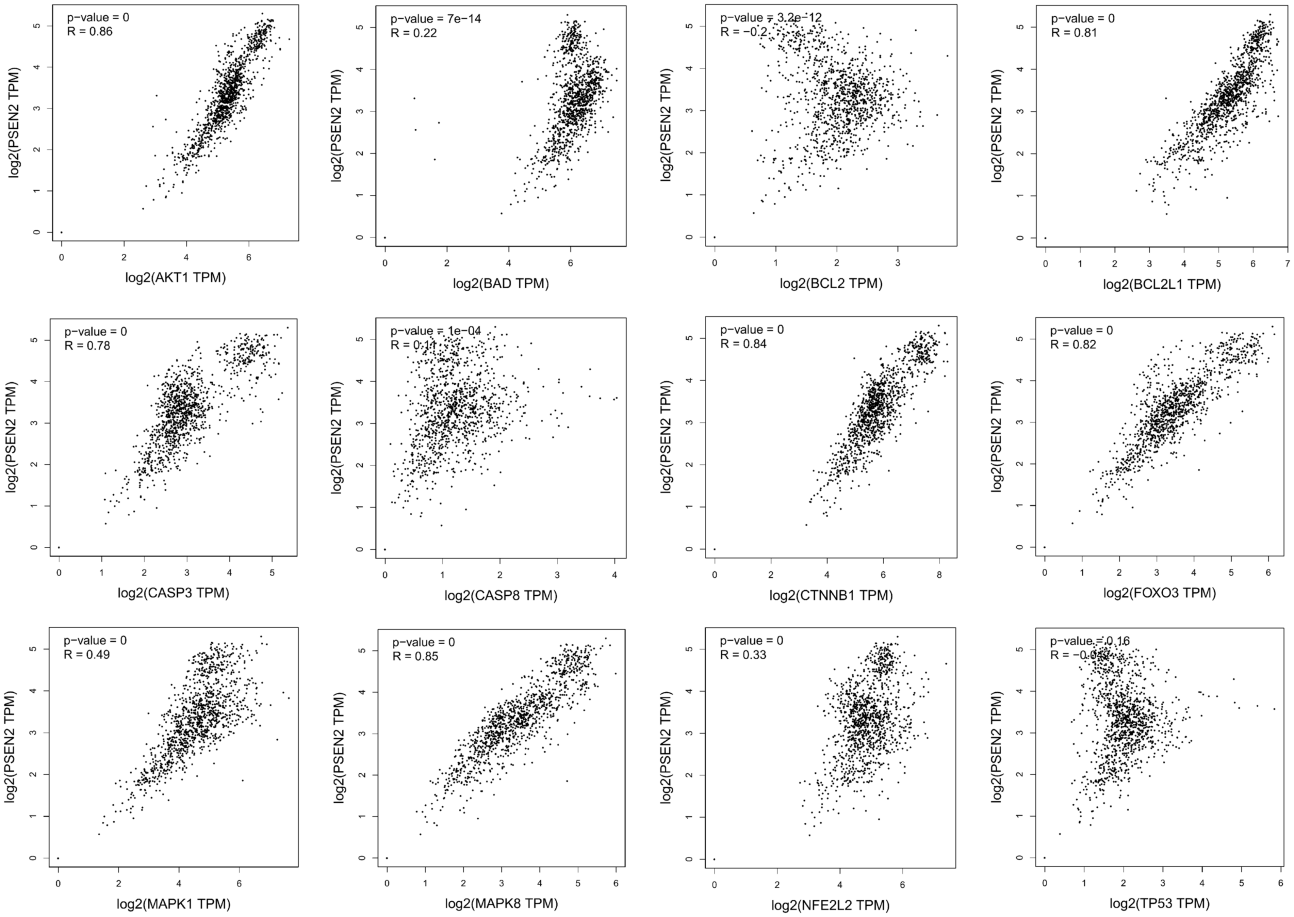
Analysis of the correlation between 12 key targets of Cordycepin and the pathogenic gene of Alzheimer’s disease PSEN2.

Missense mutation of APP gene changes A β Metabolize and regulate A β And cause A β Cognitive decline (Lan et al., 2014). To confirm the relationship between key genes and major causative gene APP, MAPT, or PSEN2, gene correlation analysis was conducted using GEPIA. Four indexes were significantly correlated with APP, 7 with PSEN2, and 8 with MAPT (Figures 5–7). It is well-established that aging accelerates and promotes cognitive disorders and is the most prominent risk factor for neurodegenerative diseases, including AD (Hou et al., 2019). We obtained 8 metrics correlated with age through analysis of the Aging Altas database (Table 1). The results showed that the five targets of AKT1, MAPK8, BCL2L1, FOXO3 and CTNNB1 were not only significantly related to APP, PSEN2 and MAPT but also related to age. Accordingly, we speculate that these five targets mediate the preventive and therapeutic effects of Cordycepin against AD (Figures 5–7).

**Table 1:** Core targets of Cordycepin-anti-Alzheimer and pathways related to aging.

Symbol	Description	Function	Gene_Set	Literature_Name	KEGG
AKT1	v-akt murine thymoma viral oncogene homolog 1	AKT1 is one of 3 closely related serine/threonine-protein kinases (AKT1, AKT2 and AKT3) called the AKT kinase that regulates many processes, including metabolism, proliferation, cell survival, growth and angiogenesis.	cellular senescence	Akt negatively regulates the in vitro lifespan of human endothelial cells via a p53/p21-dependent pathway	Longevity regulating pathway
TP53	tumor protein p53	TP53 acts as a tumor suppressor in many tumor types; induces growth arrest or apoptosis depending on the physiological circumstances and cell type. TP53 is involved in cell cycle regulation as a trans-activator that acts to negatively regulate cell division by controlling a set of genes required for this process.	others	Variation in the human TP53 gene affects old age survival and cancer mortality	Longevity regulating pathway
MAPK8	mitogen-activated protein kinase 8	MAPK8, also known as JNK1, encodes many transcripts and is an important player in stress response. Overexpression of JNK in roundworms also increases lifespan.	deregulated nutrient sensing	JNK Extends Life Span and Limits Growth by Antagonizing Cellular and Organism-Wide Responses to Insulin Signaling	Insulin signaling pathway
BCL2L1	BCL2 like 1	Potent inhibitor of cell death. Inhibits activation of caspases. Appears to regulate cell death by blocking the voltage-dependent anion channel (VDAC) by binding to it and preventing the release of the caspase activator, CYC1, from the mitochondrial membrane.	NF-κB related gene	Human exceptional longevity: transcriptome from centenarians is distinct from septuagenarians and reveals a role of Bcl-xL in successful aging	NF-kappa B signaling pathway
FOXO3	forkhead box O3	A transcription factor of the Fox family, FOXO3, is crucial in development.	others	Fox’s in Development and Disease	Longevity regulating pathway
BCL2	B-cell CLL/lymphoma 2	Suppresses apoptosis in various cell systems, including factor-dependent lymphohematopoietic and neural cells. Regulates cell death by controlling the mitochondrial membrane permeability.	others	Regulation of apoptosis resistance and ontogeny of age-dependent diseases	p53 signaling pathway
CTNNB1	catenin (cadherin-associated protein), beta 1, 88 kDa	CTNNB1, also known as beta-catenin, is a member of the adherens junctions proteins, involved in epithelial layers that mediate adhesion between cells, cell communication, growth, embryogenesis, and wound healing.	altered intercellular communication	beta-Catenin Controls Hair Follicle Morphogenesis and Stem Cell Differentiation in the Skin	Wnt signaling pathway
NFE2L2	nuclear factor, erythroid 2-like 2	NFE2L2 is a transcription factor that plays a key role in the response to oxidative stress: The NFE2L2/NRF2 pathway is also activated in response to selective autophagy: autophagy promotes interaction between KEAP1 and SQSTM1/p62 and subsequent inactivation of the BCR(KEAP1) complex, leading to NFE2L2/NRF2 nuclear accumulation and expression of cytoprotective genes.	genomic instability	p62/SQSTM1 Is a Target Gene for Transcription Factor NRF2 and Creates a Positive Feedback Loop by Inducing Antioxidant Response Element-Driven Gene Transcription	Unknown

### Molecular docking

**Figure 8:**
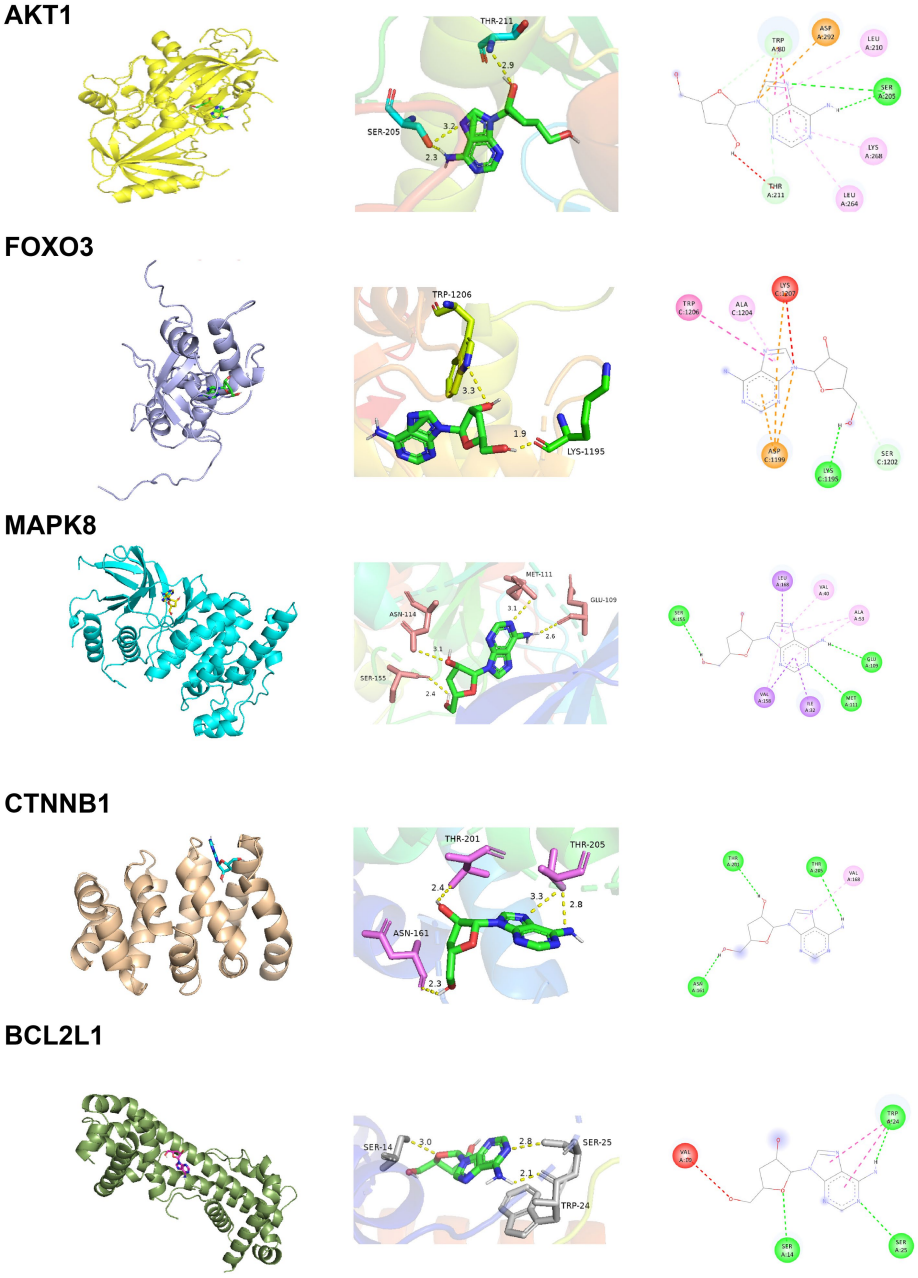
Docking results of Cordycepin with the core target.

The docking energy associated with the binding of Cordycepin to the core target was calculated using the AutoDock Vina software. It has been established that the target possesses significant binding capacity with the compound when the docking energy is less than −1.2 kcal/mol or −5 kJ/mol. In our study, the binding energy between 5 core targets and Cordycepin were less than −5 kJ/mol, respectively (Table 2), which indicates that the ligand can spontaneously bind to the receptor molecule. These results suggested that these targets might play an important role in Cordycepin treatment of AD. The specific docking parameters are shown in Figure 8.

**Table 2:** Molecular docking parameters of cordycepin core targets.

Target	PDB ID	Coordinate	Binding energy (kJ/mol)	Cordycepin docking amino acid residues
AKT1	4ejn	x = 29.817; y = 43.243; z = 15.125	−7.8	Chain A: ASN53 ASN54 PHE55 SER56 VAL57 ALA58 GLN59 CYS60 CYS77 LEU78 GLN79 TRP80 VAL270
BCL2L1	6uvg	x = 87.133; y = −4.627; z = 136.989	−7	Chain A: GLU7 VAL10 ASP11 SER14 TYR15 SER18 TYR22 SER23 TRP24 SER25 MET83 ALA84 ALA85 LYS87 GLN88 ARG91; Chain B: ASP11 LYS87 ARG91
MAPK8	3elj	x = 21.284; y = 16.240; z = 29.260	−6.8	Chain A: ILE32 GLY33 SER34 GLY35 VAL40 ALA53 ILE86 MET108 GLU109 LEU110 MET111 ASP112 ASN114 SER155 ASN156 VAL158 LEU168
FOXO3	2uzk	x = 14.088; y = −19.100; z = 39.088	−6.6	Chain C: SER1181 TYR1184 GLU1185 VAL1188 PHE1194 LYS1195 ASP1196 LYS1197 GLY1198 ASP1199 SER1202 SER1203 ALA1204 TRP1206 LYS1207
CTNNB1	7afw	x = 65.770;y = −43.227; z = 28.298	−6.2	Chain A: LEU160 ASN161 ASP162 GLN165 VAL168 ALA197 ARG200 THR201 ASN204 THR205 ASN206 ASP207

### Analysis of core gene expression

**Figure 9:**
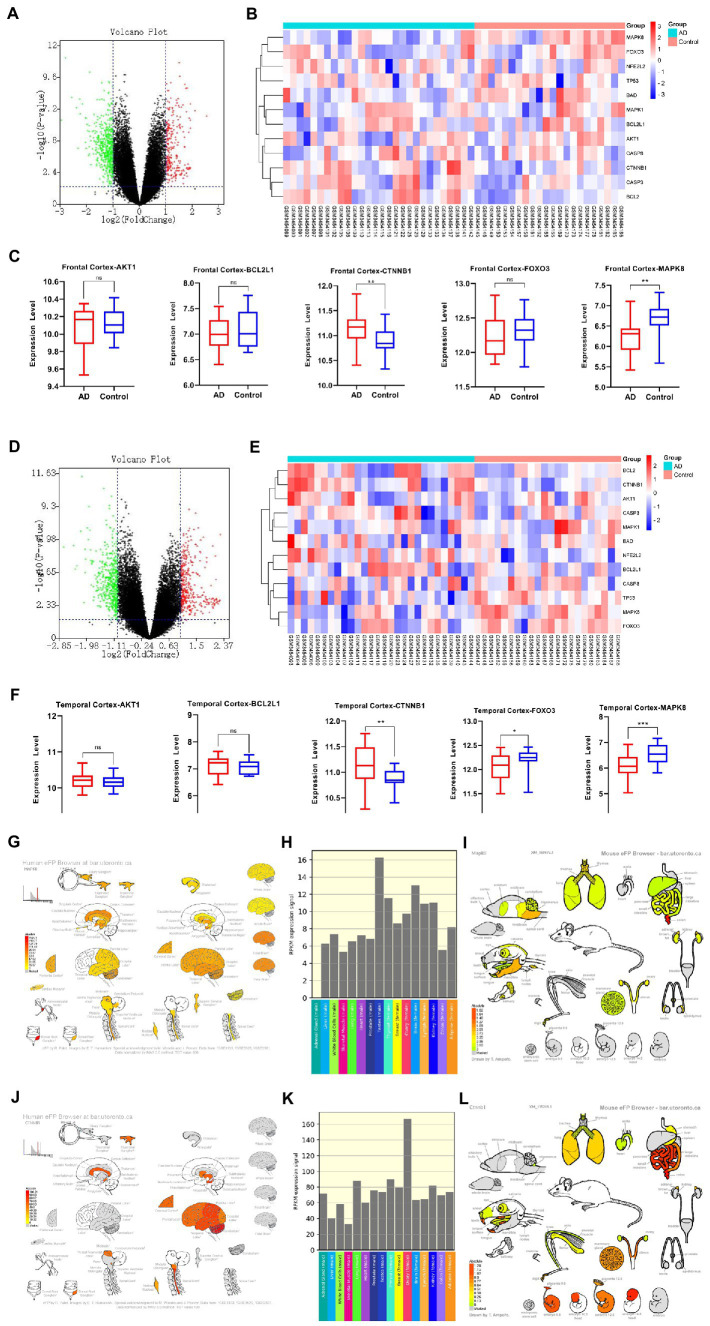
AD mRNA Expression analysis using GEO Dataset **(A,C,D,F)**. Composition-Target binding energy heat map **(B,E)**. MAPK8 expression analysis in the regular human nervous system **(G,H)**. MAPK8 expression analysis in tissues and organs of a normal mouse **(I)**. CTNNB1 expression in the normal human nervous system **(J,K)**. CTNNB1 expression analysis in tissues and organs of normal mouse **(L)**. *p < 0.05, **p < 0.01.

The AD brain transcriptome GSE122063 dataset, which examined the expression of two different brain regions, frontal cortex (FC) and temporal cortex (TC) in AD (*n* = 12) and normal controls (*n* = 11), was used to evaluate the expression changes of 5 core targets in brain tissue of AD patients. Among AD patients, the mRNA expression of CTNNB1 was significantly increased in TC and FC tissues, while MAPK8 was significantly downregulated. In contrast, FOXO3 was significantly reduced only in TC tissues. There was no significant difference in BCL2L1 and AKT1 levels between TC and FC tissues (Figures 9A–F). The expression of MAPK8 and CTNNB1 in the normal nervous system was analyzed by the Human eFP Browser. As shown in Figures 9G–L, MAPK8 was expressed in the whole human nervous system in healthy subjects, with the highest expression in male testis and the colon, mandible and hindbrain of mice. The expression of CTNNB1 was the highest in the cerebellum of the nervous system and the highest in the human female ovary. It was mainly distributed in mouse colon, embryonic development and teeth.

## Discussion

In this article, we analyzed the multi-level mechanism of Cordycepin in treating AD. The intersection of active component targets and disease-related targets yielded 74 common targets. Twelve targets were identified by PPI network analysis, while 5 targets AKT1, MAPK8, BCL2L1, FOXO3 and CTNNB1 were positively correlated with AD pathogenic genes APP, PSEN2 and MAPT, and associated with aging genes Our findings suggest that MAPK8, FOXO3 and CTNNB1 have clinical research value. GO annotation and KEGG pathway analysis were conducted. As shown in Figures 3A,B, significantly enriched BP terms mainly included positive and negative regulation of apoptosis, neuron apoptosis and drug response, MF terms included participation in enzyme activity, protease and transcription factor binding, and CC terms included nucleus, macromolecular complex and mitochondria. The enriched GO items were also associated with neuronal apoptosis, death-induced signal complex formation, cysteine endopeptidase activity, and so on.

AD is considered to be a continuous and gradual process, about 25 years from the onset of pathology to death. Amyloid initiates this process, which is followed by a variety of neurobiological processes, including tau pathology, brain atrophy and synaptic dysfunction, initially with a slight cognitive decline, followed by memory impairment, aphasia, visual–spatial skills impairment, executive dysfunction, personality and behavioral changes (Aisen et al., 2022). Two neuropathological features of AD are extracellular deposition ofβ-amyloid (Aβ) peptide and intracellular accumulation / deposition of hyperphosphorylated tau protein (Montine et al., 2011; Hyman et al., 2012). The study found that although amyloid protein levels were similar, people with strong resilience to AD had lower levels of hyperphosphorylated tau accumulation in synapses and neocortical regions (Van Rossum et al., 2012). There are many genetic changes in AD disease, and many genes are involved in the process of remission and aggravation of the disease (Hansen et al., 2017; Yu et al., 2020). Amyloid precursor protein (APP) is a kind of intramembrane protein expressed in various tissues, which is concentrated in the synapses of neurons. It can bind to death receptor 6 to initiate apoptosis and further induce neuronal death (Michaud et al., 2013; Xu et al., 2022).The hyperphosphorylation and accumulation of microtubule-associated protein tau (MAPT) forms the initial event before neurodegeneration (Lee et al., 2001). In humans, neuroinflammation is positively correlated with tau pathology and participates in tau hyperphosphorylation, accumulation and neurodegeneration (Zilka et al., 2009). Of the 12 core targets obtained in this study, 7 were significantly correlated with MAPT, indicating that they were more closely related to tau lesions.

**Figure 10:**
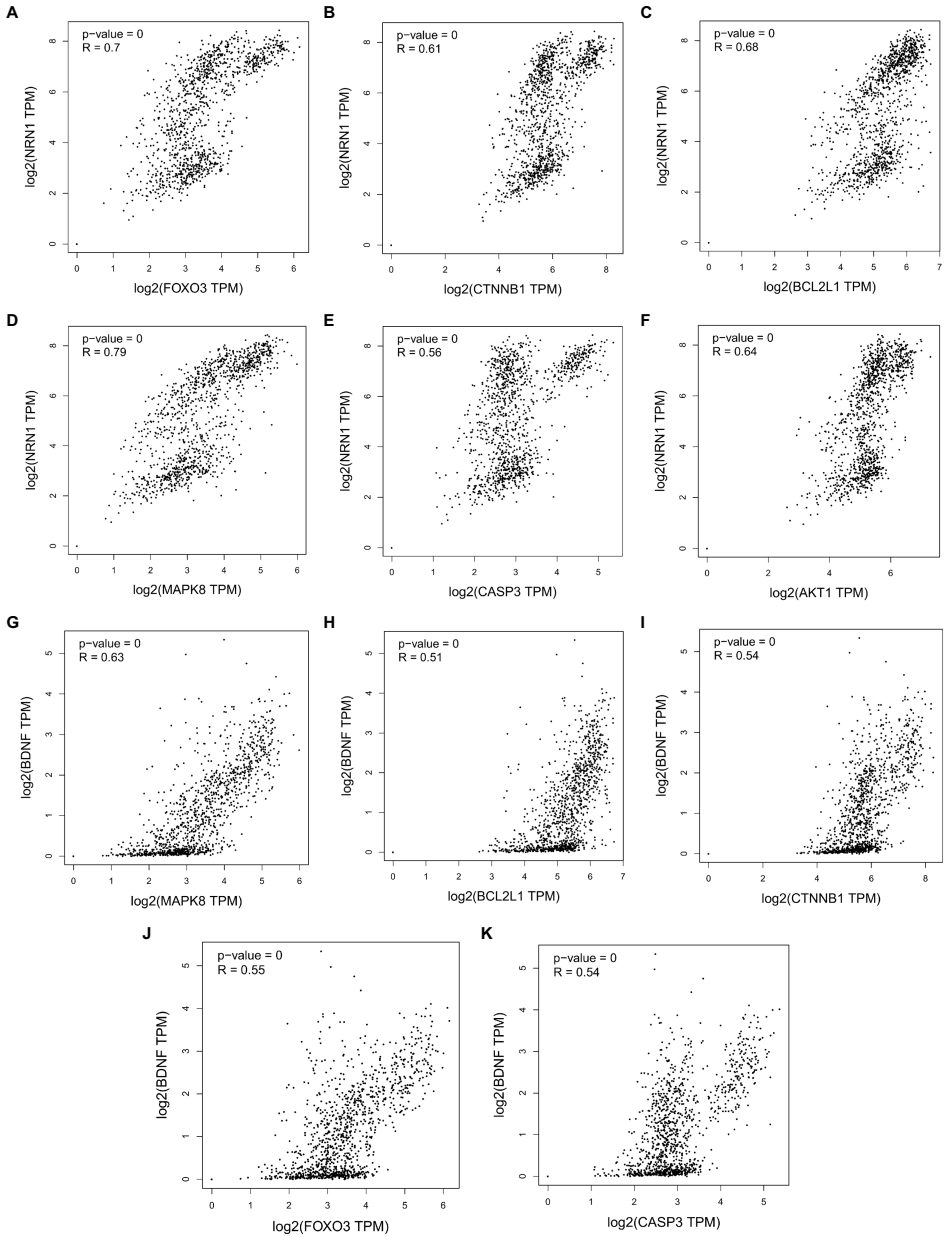
Correlation analysis between cordycepin key targets and neurotrophin NRN1 **(A–F)**; correlation analysis between cordycepin key targets and neurotrophin BDNF **(G–K)**.

The growing evidence that both Aβ and tau lesions are powerful causes of cell aging Senescent cells have been detected in the brains of patients with AD. (Gebicke-Haerter, 2001; Baker et al, 2018; Zhang et al, 2019; Han et al, 2020; SaezAtienzar et al, 2020). The removal of aging cells by drug and genetic methods can reduce the brain Aβ load and tau disease, and improve the memory (Bussian et al., 2018; Musi et al., 2018; Zhang et al., 2019; Tam and Ju, 2022) of these AD model mice, Not only that, the study found that people with strong resilience to AD showed a unique cytokine spectrum (McKay et al., 2019), including higher levels of anti-inflammatory cytokines and neurotrophins, as well as lower levels of chemokines (Perez-Nievas et al., 2013; Barroeta-Espar et al., 2019). Initial studies have identified neurotrophic factors including NRN1 and BDNF that may contribute to the recovery of AD pathology (Neuner et al., 2022). As neurotrophic factors, they play an important role in synaptic function and plasticity as well as in maintaining axonal morphology (Yao et al., 2018). It is worth noting that the five core targets of cordycepin in alleviating AD obtained in this study were significantly correlated with the expression of NRN1 and BDNF except AKT1 (Figure 10). This further indicates that cordycepin can increase the expression of neurotrophic factors.

We have identified five target genes which were potential core targets mediating the therapeutic effect of Cordycepin against AD in this study. There is compelling evidence that the activity of β-and γ-secretase can regulate MAPK signal pathway (Salminen et al., 2016; Sochocka et al., 2017; Benito-Cuesta et al., 2020), neuronal apoptosis and phosphorylation of APP and Tau participate in the pathogenesis of AD (Kim and Choi, 2010). The anti-apoptotic protein BCL2L1 regulates the death of β-apoptotic cells through Bcl-2 proteins. Interestingly, it has been shown that increased expression of BCL2L1 is related to the chemotherapy resistance of T-ALL (Broome et al., 2002). β-catenin signals encoded by CTNNB1 regulate a variety of different pathways in the pathogenesis of AD, such as synaptic plasticity, neuronal survival, inflammation and tau phosphorylation (Jia et al., 2019). AD is an age-related disease, and the activation of FOXO3 has been found to improve somatic cell maintenance and prolong life. As a transcription factor, FOXO3 plays a central role in cellular stress and contributes to redox regulation, autophagy, energy homeostasis, DNA repair, cell cycle arrest, telomere maintenance and stem cell homeostasis (Davy et al., 2018). Recent studies have shown that the PI3K-Akt signal pathway may be an important target for AD therapy. Moreover, the PI3K-Akt pathway regulates many biological processes such as cell proliferation, movement, growth, survival and metabolism and inhibits different neurotoxic mechanisms (Kumar and Bansal, 2022). In addition, PI3K/Akt facilitates the activation of neurons and neural stem cells (Razani et al., 2021).

**Figure 11:**
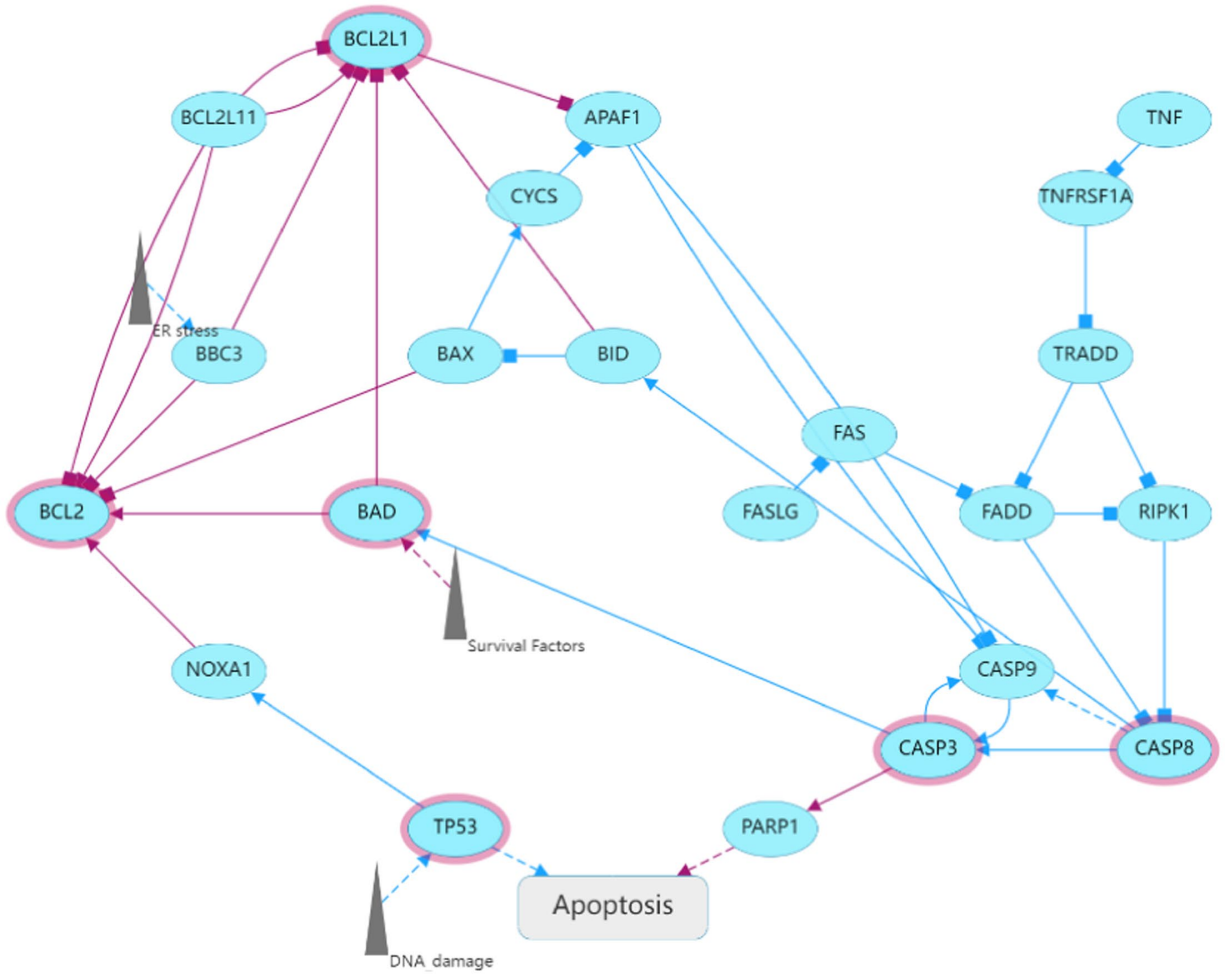
Key genes of cordycepin against AD involved in apoptosis.

Cordycepin is a metabolite with antiviral activity produced by fungal Cordyceps Militaris. It has a variety of pharmacological functions, including immunomodulatory, neuroprotective and anti-inflammatory activities, and even inhibits tumorigenesis and cancer development (Wang et al., 2019). Interestingly, we found that cordycepin was first extracted from fungi of Cordyceps, but not only present in Cordyceps, but some have also detected genes that synthesize pentostatin and cordycepin from Aspergillus (Xia et al., 2017). This perhaps suggests that similar mechanisms of active ingredient synthesis may exist for different fungi. In a Parkinson’s disease model, Cordycepin was found to relieve motor disorders and exert a neuroprotective role (Cheng and Zhu, 2019) by reducing inflammation and oxidative stress. Overwhelming evidence substantiates that Cordycepin can weaken the inflammatory response and the production of pro-apoptotic proteins, increase the expression of anti-apoptotic proteins, and inhibit the activation of the MAPK/NF- κB signaling pathway (Ding et al., 2022), consistent with the findings of this study (Figure 11). It has been established that Cordycepin can bind to the cysteine endopeptidase of the caspase family in mitochondria to inhibit the formation of the death-induced signal complex of endopeptidases recruited in the death domain and then affect the activation of apoptosis and slow down the death of neurons. Moreover, the five core targets of Cordycepin are significantly related to MAPT. Among these, AKT1, BCL2L1, and MAPK8 exhibit a high linear correlation with the expression of MAPT. Besides, AKT1, MAPK8, BCL2L1, FOXO3, and CTNNB1 exhibited a high linear correlation with PSEN2 expression, indicating that they were more closely related to tau lesions and PSEN2 expression. Finally, correlation analysis found that the five core targets were significantly correlated with the expression of neurotrophic factors NRN1 and BDNF. The above results suggest that Cordycepin can slow down neuronal apoptosis and inhibit inflammation. Moreover, Cordycepin can affect pathological tau formation and increase the expression of neurotrophic factors and maintain synaptic function.

## Conclusion

Taken together, Cordycepin exhibits multitarget characteristics during AD therapy providing novel insights for developing an optimal treatment for this patient population. Importantly, in this work, we harnessed pharmacologic strategies to investigate the therapeutic mechanisms and potential disease therapeutic mechanisms of Cordycepin for the prevention and treatment of Alzheimer’s disease. Cordycepin is expected to be a potential active ingredient for treating AD. However, further studies are warranted to increase the robustness of our findings.

## Data availability statement

Publicly available datasets were analyzed in this study. This data can be found here: GSE122063.

## Author contributions

XM designed the paper. JX revised the paper. All authors contributed to the article and approved the submitted version.

## Funding

This work was supported by the Department of Science & Technology of Liaoning Province (2021JH1/10400035) and Applied Basic Research Program of Liaoning Province 2022JH2/101300160.

## Conflict of interest

The authors declare that the research was conducted in the absence of any commercial or financial relationships that could be construed as a potential conflict of interest.

## Publisher’s note

All claims expressed in this article are solely those of the authors and do not necessarily represent those of their affiliated organizations, or those of the publisher, the editors and the reviewers. Any product that may be evaluated in this article, or claim that may be made by its manufacturer, is not guaranteed or endorsed by the publisher.

## Footnotes

^1^ http://tcmspw.com/tcmsp.php

^2^ https://www.ncbi.nlm.nih.gov/gene

^3^ http://www.symmap.org/

^4^ http://ctdbase.org/

^5^ https://www.genecards.org/

^6^ https://www.disgenet.org/

^7^ http://ctdbase.org/

^8^ https://bioinfogp.cnb.csic.es/tools/venny/

^9^ https://david.ncifcrf.gov/

^10^ http://www.bioinformatics.com.cn

^11^ http://string-db.org

^12^ https://www.rcsb.org/

^13^ http://bar.utoronto.ca/mouse_efp/cgi-bin/efpWeb.cgi

^14^ https://string-db.org/
